# Overexpression of calmodulin-like (*ShCML44*) stress-responsive gene from *Solanum habrochaites* enhances tolerance to multiple abiotic stresses

**DOI:** 10.1038/srep31772

**Published:** 2016-08-22

**Authors:** Shoaib Munir, Hui Liu, Yali Xing, Saddam Hussain, Bo Ouyang, Yuyang Zhang, Hanxia Li, Zhibiao Ye

**Affiliations:** 1 Key Laboratory of Horticultural Plant Biology (Ministry of Education), College of Horticulture and Forestry Sciences, Huazhong Agricultural University, Wuhan 430070, China; 2 Key Laboratory of Biology and Genetic Resources of Rubber Tree, Ministry of Agriculture, Rubber Research Institute, Chinese Academy of Tropical Agricultural Sciences, Danzhou 571737, China; 3 College of Resources and Environment, Huazhong Agricultural University, Wuhan 430070, China

## Abstract

Calmodulin-like (CML) proteins are important Ca^2+^ sensors, which play significant role in mediating plant stress tolerance. In the present study, cold responsive calmodulin-like (*ShCML44*) gene was isolated from cold tolerant wild tomato (*Solanum habrochaites*), and functionally characterized. The *ShCML44* was differentially expressed in all plant tissues including root, stem, leaf, flower and fruit, and was strongly up-regulated under cold, drought and salinity stresses along with plant growth hormones. Under cold stress, progressive increase in the expression of *ShCML44* was observed particularly in cold-tolerant *S. habrochaites.* The *ShCML44-*overexpressed plants showed greater tolerance to cold, drought, and salinity stresses, and recorded higher germination and better seedling growth. Transgenic tomato plants demonstrated higher antioxidant enzymes activity, gas exchange and water retention capacity with lower malondialdehyde accumulation and membrane damage under cold and drought stresses compared to wild-type. Moreover, transgenic plants exhibited reduced reactive oxygen species and higher relative water contents under cold and drought stress, respectively. Greater stress tolerance of transgenic plants was further reflected by the up-/down-regulation of stress-related genes including *SOD*, *GST*, *CAT*, *POD*, *LOX*, *PR* and *ERD.* In crux, these results strengthen the molecular understanding of *ShCML44* gene to improve the abiotic stress tolerance in tomato.

Plant growth and development is adversely affected by various abiotic stresses including cold, drought and salinity. Abiotic stresses negatively affect the crop productivity by limiting their genetic potential^1^. Plants are prone to optimum environmental conditions, thus unexpected environmental changes like low or high temperature, drought, and salinity cause almost 50% losses in the yield of major crops^2^. Recent advancements in research have made significant progress to understand the cellular and molecular mechanisms of stress towards crop productivity. Scientists have determined different physiological and biochemical pathways to probe the plant’s stress response mechanisms^3^,^4^. In plants, sub-optimal temperature causes membrane damage and dehydration. Cold stress-induced physiological and biochemical responses lead to accumulation of compatible osmolytes and reactive oxygen species (ROS), which in turns alter the membrane lipid composition^5^. Likewise, drought and salinity stresses adversely affect the ionic and osmotic equilibrium of the cell. The former displaces the membrane proteins and results in the loss of membrane integrity, disruption of cellular compartmentalization, and alterations in enzymatic activity. Dehydration of protoplasm induces the disruption of cellular metabolism, while, salinity can cause hyperosmotic and hyperionic stress leading to plant death^2^.

It is highly encouraged to explore the stress adaptive responses at cellular and whole-plant levels in order to maintain the environmental sustainability. The bottleneck of this endeavor is to keep identifying the stress-responsive genes by genomics technologies^6^,^7^. These genes may belong to different protein groups according to their domain. Several studies have reported that abiotic stresses (cold, high salt, and drought) trigger rapid increase in plant cells calcium (Ca^2+^) levels^5^,^8^. Calcium is a universal second messenger, which regulates the responses to plant’s growth and development as well as different environmental stimuli^5^,^9^. Changes in cellular Ca^2+^ level is being mediated by different Ca^2+^ binding proteins like calmodulin (CaM) and CaM-related proteins (CML), calcium-dependent protein kinases (CDPKs) and calcineurin-B-like proteins (CBL) by binding to EF-hand domain^10^,^11^.

The CaM is a relatively small protein that only contains four EF-hands and acts as ubiquitous Ca^2+^ binding protein in plants and animals^12^. The EF-hand motif is the most frequent motif found in Ca^2+^ binding proteins, and it helps Ca^2+^ binding^13^. Despite sequence divergence in EF-hands, CMLs behave as unique Ca^2+^ sensors^13^,^14^.

CMLs are the small multifunctional proteins, which interact with a broad range of Ca^2+^ binding downstream targets and alter the activities of target proteins by transducing the signal of increased Ca^2+^. CMLs contain Ca^2+^ binding motifs and do not possess other functional domains^15^. CMLs have been identified in numerous plant species, such as 50 members in *Arabidopsis*, 48 in *thellungiella halophile*, 51 in *Eucalyptus grandis*, 56 in *Populus trichocarpa*, 46 in *Medicago truncatula*^16^, 32 in rice^17^ and 52 in tomato^14^. All the CMLs contain EF-hand motifs but no other known or identifiable functional domain. Most CMLs in *Arabidopsis* (31/50), rice (20/32) and tomato (26/52) are predicted to have four EF-hands as in CaM, but a majority of them have less than 50% similarity with CaM^13^.

Various signaling transduction pathways in plant have been found to be induced by CML proteins. It is well established that CML genes can respond to different hormonal and stress stimuli^18^,^19^,^20^,^21^,^22^. In *Arabidopsis,* salinity and salicylic acid significantly up-regulated the expression of stress-responsive *AtCML8* gene^23^. Furthermore, expression analysis revealed that *AtCML9* knockout mutants exhibited more tolerance to salinity and drought by the higher accumulation of amino acids^24^. Moreover*, AtCaM15* (also called *AtCML18*) interacts with *At*NHX1 and alters the Na^+^/K^+^ selectivity of the exchanger by decreasing its Na^+^/H^+^ exchange speed. This interaction suggests the presence of Ca^2+^ dependent signaling to mediate salt stress responses^25^. Another CML gene *AtCML24*/TCH2 was induced by cold, drought and might be involved in cold-related Ca^2+^ signals transduction^26^,^27^. A novel CML gene *OsMSR2* (*Oryza sativa* multi-stress-responsive gene2) has been reported to confer the drought and salt tolerance of plants through ABA-mediated pathways in *Arabidopsis*^3^. Promoter analysis of *AtCML37* and *AtCML38* revealed that these genes are responsive to several stimuli including salt, oxidative stress and hormonal treatments^28^. Recently, Vadassery *et al.* have reported that CML42 serves as Ca^2+^ signaling component and is involved in the abiotic stress responses^29^. Altogether, these lines of evidence indicate that CML proteins play a critical role in Ca^2+^ signaling during plant development and adaptation to abiotic stress. However, the complex mechanism of CML proteins in regulating these stress pathways is still needed to be explored.

Cultivated tomato (*Solanum lycopersicum*) is prone to be affected by environmental stresses. The wild tomato is known to share adequately rich gene pool for genetic improvement. Previously, *S. habrochaites* is declared as a potential genetic resource to enhance tomato cold tolerance^30^. It has been reported that CML44 gene is induced in cold tolerant tomato genotypes rather than in the sensitive one under cold stress^31^, suggesting that this gene may play an important role in stress mechanism and may be responsible for multiple stress tolerance in tomato.

In the present study, we reported that possible role of calmodulin-related protein *ShCML44* in tolerance to different abiotic stresses. The expression pattern of *ShCML44* was examined under multiple stress conditions. Our findings clearly indicate that overexpressed *ShCML44* gene plants exhibit significant improvement in tolerance to cold, drought and salinity stress by higher antioxidant enzymes activity, lower malondialdehyde (MDA) and membrane damage, better gas exchange and maintenance of plant water relations. Moreover, transgenic plants exhibited reduced ROS accumulation under cold stress and higher relative water contents under dehydration stress. Taken together, these finding can be used for genetically modified plants to improve the abiotic stress tolerance of tomato in future.

## Results

### Isolation and sequence analysis of ***ShCML44***

**Figure 1:**
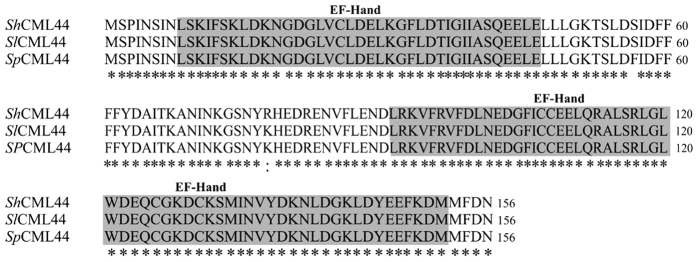
Alignment of *ShCML44* protein sequence with sequences from *S. lycopersicum* (*SlCML44*) and *S. pennellii* (*SpCML44*). Multiple sequence alignments were conducted using ClustalW2. The GenBank accession numbers for *ShCML44*, *SlCML44* and *SpCML44* protein sequences are KX011024, XM_004237307 and XM_015217409, respectively.

Previously, a microarray analysis has been performed to compare the gene expression between the cold-sensitive genotype (*S. lycopersicum* AC) and cold-tolerant genotypes (*S. habrochaites* LA1777 and an introgression line LA3969) under cold stress, and it has been found that SGN-U227216 was more strongly induced in the two tolerant genotypes than in the sensitive one^32^. This unigene is homologous to *S. lycopersicum* Ca-binding protein mRNA LOC101245247 (designated as *SlCML44*, GenBank accession no. XM_004237307) and *S. pennellii* Ca-binding protein mRNA LOC107017137 (designated as *SpCML44*, GenBank accession no. XM_015217409). Based on this sequence, the *S. habrochaites* homologous full-length cDNA sequence was isolated and designated as *ShCML44* (GenBank accession no. KX011024). The *ShCML44* ORF contains 471 bp and encoding a protein of 156 amino acids with predicted 18.04 kDa molecular weight and 4.49 pI. Alignment of the three *Solanum* deduced amino acid sequences revealed that the *ShCML44* amino acid sequence has 99% identity with the Micro-Tom CML LOC101245247 (GenBank accession no. XM_004237307) and the *S. pennellii* CML LOC107017137 (GenBank accession no. XM_015217409). Only one amino acid numbered 56, was different between *S. lycopersicum* and *S. pennellii* wild species by substituting serine (in Micro-Tom) with phenylalanine in *S. pennellii*. Similarly, only one amino acid numbered 78, was different between *S. lycopersicum* and *S. habrochaites* wild species by substituting lysine (in Micro-Tom) with arginine in *S. habrochaites* (Fig. 1). The *ShCML44* phylogenetic analysis indicated the highest homology among CMLs from other *Solanum* species including *S. tuberosum, Nicotiana sylvestri* and *Nicotiana tomentosiformis.* The *ShCML44* showed approximately 97% identity with *S. tuberosum* CML44 protein (GenBank accession no. XM_006365362), and 84% identity with both *Nicotiana sylvestri* (GenBank accession no. XM_009782235) and *Nicotiana tomentosiformis* (GenBank accession no. XM_009619341). The *ShCML44* closest *Arabidopsis* homologue is *AtCML44* (GenBank accession no. NP_564143) (Fig. S2).

### Expression pattern analysis of ***ShCML44***

**Figure 2:**
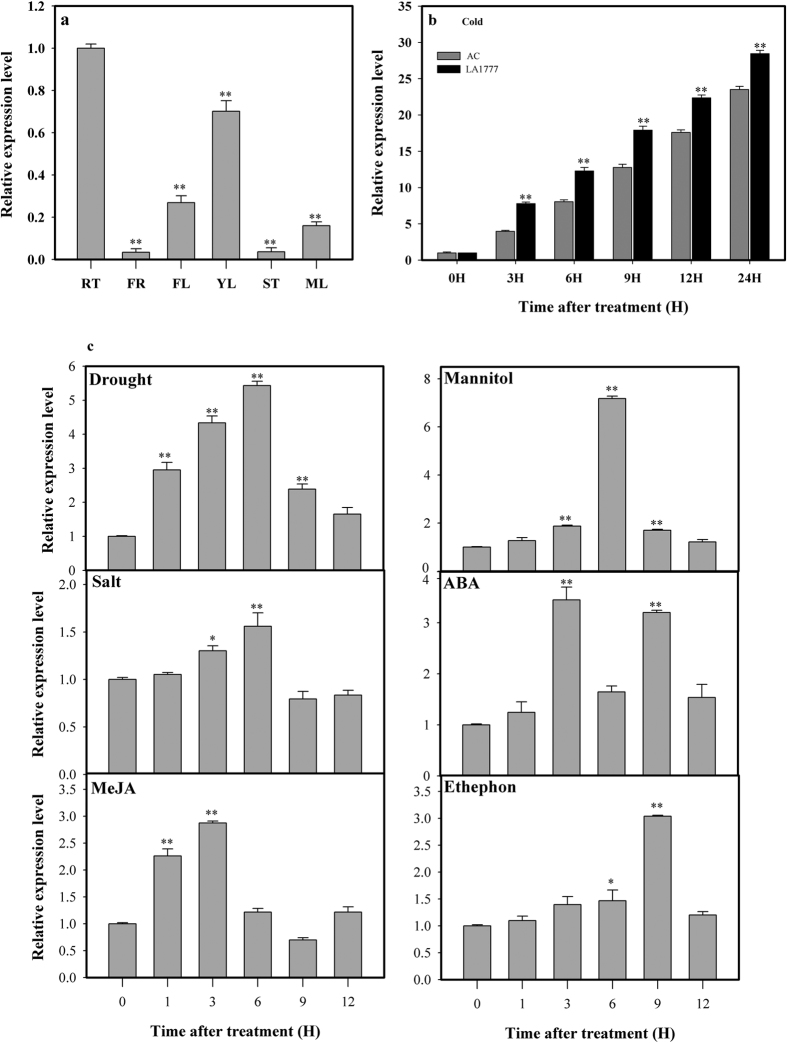
Expression patterns of CML44 under different treatments or various tissues. (**a**) Expression pattern of *ShCML44* gene in various organs (RT, root; FR, fruit, FL, flower, YL, young leaf; ST, stem; ML, mature leaf) of *S. habrochaites* LA1777 plants. Tissues were collected from six plants to perform qRT-PCR analysis. Asterisks indicate a significant difference (*P < 0.05; **P < 0.01, Dunnett’s multiple comparisons test) compared with the root. (**b**) Comparative express level analysis of **CML44 in** *S. habrochaites* LA1777 and *SlCML44* in *S. lycopersicum* AC under cold stress at 4 °C for 1, 3, 6, 9, 12, and 24 h. Zero (0) represents seedlings without any treatment. Asterisks indicate significant differences between LA1777 and AC at every time point. *P < 0.05; **P < 0.01, student’s t-test. *ShCML44* expression pattern in LA1777 seedlings under treatments of drought (dehydration), salt (200 mM NaCl), and exogenous hormones (100 μM ABA, 100 μM MeJA, 100 μM Mannitol and 1% (v/v) ethephon) for 1, 3, 6, 9, and 12 h. Zero (0) represents seedlings without any treatment. Asterisks indicate a significant difference (*P < 0.05; **P < 0.01, Dunnett’s multiple comparisons test) compared with the corresponding controls (0 h). For internal control, EF1α gene was used in qRT-PCR analysis. Values represent the means ± SE of three biological replicate samples. Each replicate sample was composite of leaves from three seedlings.

The *ShCML44* tissue-specific expression analysis was carried out using qRT-PCR. Analysis revealed that the *ShCML44* was constitutively expressed in all tissues, and the abundance accumulation was observed in leaves and flowers of *S. habrochaites* LA1777 (Fig. 2a).

For microarray data confirmation, expression analysis of *ShCML44* in the cold-tolerant *S.habrochaites* LA1777 and *SlCML44* in the cold-sensitive *S. lycopersicum* AC were carried out under cold stress using qRT-PCR. Transcripts of *ShCML44* and *SlCML44* showed time-dependent increases under cold stress. However, the relative expression level of *ShCML44* under cold stress was significantly higher than that of *SlCML44*. Exposure of cold stress for 24 h enhanced the relative expression of *ShCML44* by 28.44-fold in LA1777, while the expression of *SlCML44* transcripts in AC was increased by 23.51-fold. However, increases in the expression of *ShCML44* in LA1777 were 96.61, 52.51, 40.60, 27.02 and 20.98% higher than those of *SlCML44* in AC at 0, 3, 6, 9, 12, and 24 h, respectively. The CML transcripts showed a positive association with tolerance to cold stress (Fig. 2b).

To further investigate *ShCML44* role in abiotic stresses, the transcript regulation of *ShCML44* in LA1777 was analyzed under various abiotic stresses and hormonal treatments. Analysis revealed that the *ShCML44* transcript levels were up-regulated under drought (dehydration), salinity, ABA, mannitol, methyl jasmonate (MeJA) and ethephon treatments. Increased *ShCML44* expression level was induced by drought, mannitol, and salinity treatments, the maximum expressions were observed at 6 h after stress exposure. The ABA and MeJA treatments also triggered the expression level of *ShCML44*, the maximum levels were observed at 3 h after treatment. The expression level of *ShCML44* was smoothly up-regulated under ethephon, the maximum at 9 h after treatment (Fig. 2c).

### Putative cis-acting regulatory elements in CML44 promoters region

To further understand the cold stress tolerance mechanism, promoter sequence (approximately 1500 bp upstream the transcription start site) was analyzed to find out the putative cis-acting regulatory elements. *S. habrochaites* promoter sequence was analyzed, and putative *cis*-acting regulatory were identified by PlantCARE^32^ and PLACE database^33^. As shown in Table 1, different stress and hormone responsive *cis*-acting regulatory elements were found, including cold related elements (LTRECOREATCOR15 and MYCCONSENSUSAT), dehydration responsive (ABRELATERD1, ACGTATERD1, MYB1AT, and MYBCORE), ABA-related elements (RYREPEATBNNAPA and ABRERATCAL), ethylene responsive (ERELEE4), gibberellin regulation element (GAREAT, MYBGAHV and TATCCAOSAMY), auxin responsive elements (ARFAT, ASF1MOTIFCAMV and NTBBF1ARROLB) and heat shock responsive (CCAATBOX1). The presence of these *cis*-elements suggests that *ShCML44* gene is actively involved in plant growth and development under stress conditions.

### Transformation and characterization of transgenic plants

To investigate the *ShCML44* functional significance and physiology effect on transgenic plants under stress treatments, plant binary expression vector pMV was used to clone *ShCML44* cDNA full-length ORF under the promoter CaMV35S control. This construct transformation was executed into tomato cultivar AC. A total number of 25 independent kanamycin-resistant (T_0_ generation) were generated, and PCR analysis was carried out to confirm the positive transformants. Further PCR analysis confirmed the 20 positive transformants using CaMV35S forward and gene-specific reverse primer pairs. Among them, 15 exhibited kanamycin resistance as 3:1 segregation ratio in the T_1_ generation, which possibly because of plants protecting a single T-DNA insert (data not shown) were designated for further CML gene expression level analysis.

To analyze the transgene expression, qRT-PCR was performed and wild-type plants expression level (wild-type = 1.0) was used to normalize the relative expression levels. We designed universal qRT-PCR primer (CML, Table S1) for both *ShCML44* and *SlCML44* because they shared high identity in nucleic acid sequences (Fig. S1). Hence, CML relative expression level in each transgenic line was the combination of *ShCML44* and *SlCML44* expression level. For further investigations, three independent transgenic lines C39, C65, C75 with the higher expression level of *ShCML44* and *SlCML44* were selected.

### Cold tolerance of ***ShCML44*** overexpressing plants

**Figure 3:**
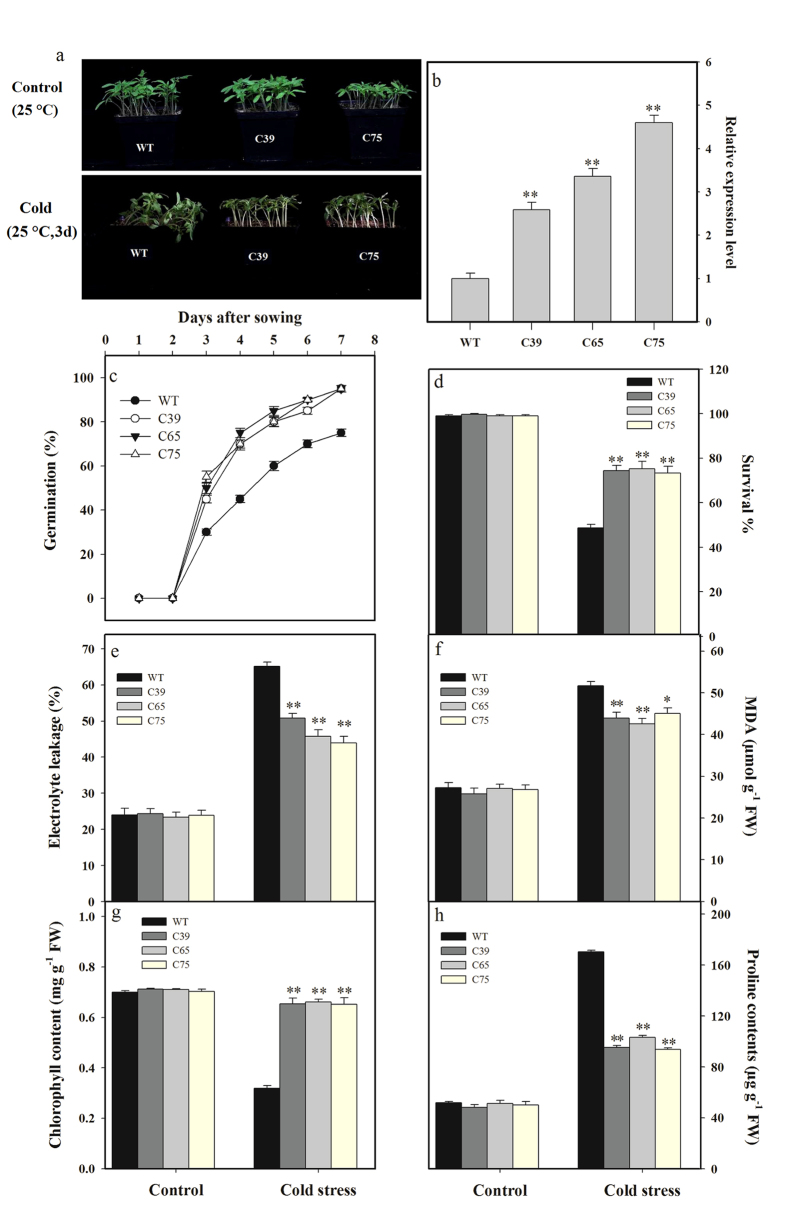
*ShCML44* overexpression plants response towards cold tolerance in tomato. (**a**) Phenotypic evaluation of transgenic lines and wild-type under normal and cold stress conditions. Two-week-old seedlings of transgenic lines (T_2_ generation) and wild-type were treated under cold 4 or 25 °C (control) for 3 d. (**b**) *SlCML44* relative expression in wild-type and *SlCML44* and *ShCML44* combined expression in transgenic lines. The relative expression levels were normalized to the wild-type expression level, which is considered as 1.0, The EF1α gene was used as an internal control. (**c**) Seed germination of transgenic lines and wild-type plants under cold stress. Seed of transgenic T_2_ lines and wild-type were treated under cold 4 or 25 °C (control) for 7 days. Comparison of survival rate (**d**), relative electrolyte leakage (**e**), MDA (**f**), chlorophyll (**g**) and proline (**h**) contents in leaves of transgenic lines and wild-type plants under normal and cold stress conditions. Values in (**c,d**) represent the means ± SE of three biological replicate samples. Each replicate sample was a composite of twenty seedlings. Values in (**e–h**) represent the means ± SE of three biological replicate samples. Each replicate sample was composite of leaves from twelve seedlings. C39, C65, and C75 represent three independent *ShCML44* transgenic tomato lines. WT represents the wild-type AC. Asterisks indicate significant differences between transgenic lines and wild-type. *P < 0.05; **P < 0.01, Dunnett’s multiple comparisons test.

To investigate the role of *ShCML44* in enhancing tolerance to cold stress, transgenic plants of three lines (T_2_ generation) along with WT (AC) were evaluated under cold stress treatment. No significant differences in growth and morphology of transgenic and wild-type plants were observed under normal growth conditions. However, 3 d exposure to cold stress (4 °C) caused wilting in the tomato seedlings particularly in the wild-type. The *ShCML44* gene expression was positively associated with the increased cold tolerance (Fig. 3a,b). Our results indicated a significant correlation between gene expression levels and cold tolerance.

The rate of germination in transgenic lines and wild-type plants was determined at 4 °C for 7 d. Results indicated a significantly higher germination in transgenic lines under cold stress than that of wild-type plants (Fig. 3c). Survival percentage was observed in transgenic and wild-type plants at 4 °C for 3 d and control (25 °C) condition. Cold stress reduced the survival percentage of tomato seedlings, however, the transgenic lines showed significantly higher (52.70%) survival percentage compared with wild-type plants (Fig. 3d).

The relative electrolyte leakage, MDA, chlorophyll and proline contents remained similar in transgenic and wild-type plants under control (25 C) temperature (Fig. 3e–h). Nonetheless, the cold-stressed seedlings exhibited prominent proliferation in relative electrolyte leakage and MDA contents. The transgenic lines showed significantly lower electrolyte leakage and MDA content than wild-type, and these attributes were well linked with the gene expression level (Fig. 3e,f). Exposure of cold stress increased the relative electrolyte leakage by 2.72-fold in wild-type and only 2.09-, 1.96- and 1.84-fold enhancements were observed in C39, C65, and C75 transgenic lines, respectively (Fig. 3e). Cold stress treatment (4 °C) induced an almost 1.90-fold increase in MDA content in wild-type and a 1.70-, 1.57- and 1.68-fold increase in C39, C65, and C75 transgenic lines, respectively (Fig. 3f). The total chlorophyll contents in transgenic lines remained unaffected by cold stress, however, the wild-type plants showed significantly lower (54.26%) accumulation of chlorophyll contents under cold stress (Fig. 3g). Cold stress induced the levels of proline in tomato seedlings, but such an increase was considerably lower in the transgenic lines than in the wild-type. In comparison with control, proline concentrations under cold stress were increased by 3.30-, 1.98-, 2.01- and 1.87-fold in the wild-type, C39, C65, and C75, respectively (Fig. 3h).

**Figure 4:**
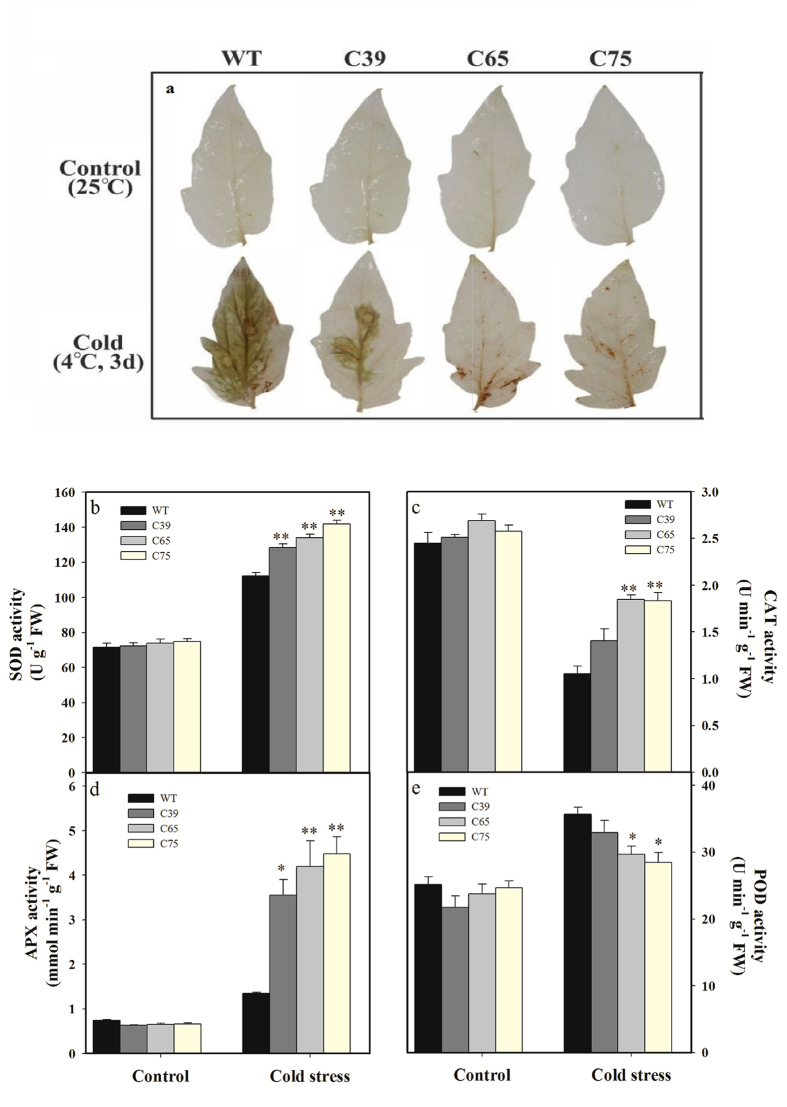
Comparison of ROS accumulation and enzymatic activity in transgenic and wild-type plants under normal and cold stress conditions. (**a**) DAB staining of H_2_O_2_ accumulation in leaves. Comparison of SOD (**b**), CAT (**c**), APX (**d**), and POD (**e**) activities in the leaves of transgenic and wild-type plants under normal and cold stress conditions. Six-week-old seedlings of transgenic lines (T_2_ generation) and wild-type were treated under cold 4 or 25 °C (control) for 3 d. For staining fully expanded terminal leaflet of upper leaves were detached from transgenic and wild-type plants. Leaves dark brown regions shown H_2_O_2_ generation. The samples shown are representative of six replicates. Values in (**b–e**) represent the means ± SE of three biological replicate samples. Each replicate sample was composite of leaves from twelve seedlings. C39, C65, and C75 represent three independent *ShCML44* transgenic tomato lines. WT represents the wild-type AC. Asterisks indicate significant differences between transgenic lines and wild-type. *P < 0.05; **P < 0.01, Dunnett’s multiple comparisons test.

To detect the ROS accumulation, hydrogen peroxide (H_2_O_2_) staining in leaves of transgenic lines and wild-type plants was carried out; the wild-type leaves showed more staining compared to transgenic lines after 4 °C for 3 d of cold stress (Fig. 4a).

To understand role of *ShCML44* in cell protection subjected to cold-induced oxidative stress damage, antioxidant enzymes (SOD, CAT, APX, and POD) activity was examined. There were no significant difference between transgenic lines and wild-type regarding antioxidant enzymes activity under normal conditions (Fig. 4b–e). Under cold stress (4 °C for 3 d) treatment, the activities of SOD, POD and APX were significantly increased, while CAT activity showed a substantial decrease in both wild-type and transgenic lines. The transgenic lines demonstrated 19.92, 61.54, and 201.80% higher activities of SOD, CAT, and APX than in wild-type, respectively (Fig. 4b–d), whereas, POD activity was decreased by 72.98% in transgenic lines (Fig. 4e).

**Figure 5:**
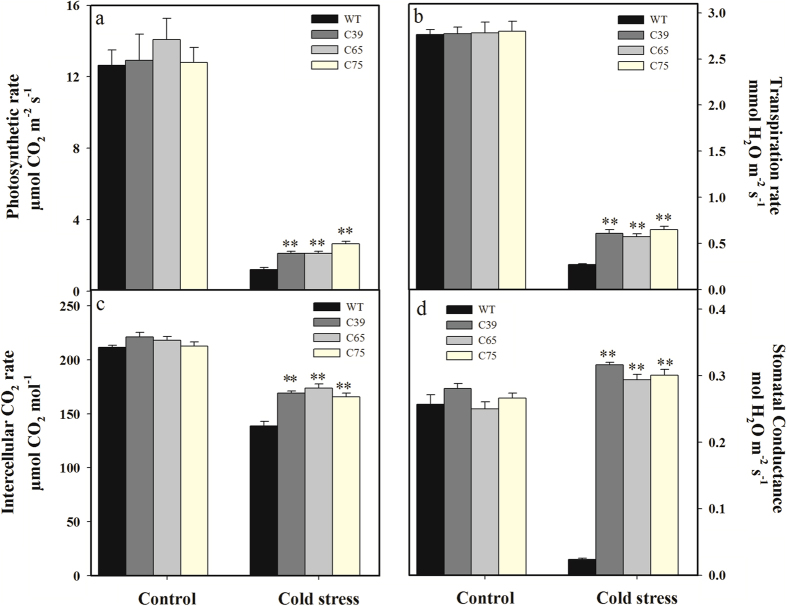
Physiological analysis of transgenic and wild-type plants under normal and cold stress conditions. Comparison of photosynthesis (**a**), transpiration (**b**), intercellular CO_2_ rate (**c**) and stomatal conductance (**d**) in the leaves of transgenic and wild-type plants under normal and cold stress conditions. Six-week-old seedlings of transgenic lines (T_2_ generation) and wild-type were treated under cold 4 or 25 °C (control) for 3 d. Values in (**a–d**) represent the means ± SE of three biological replicate samples. Each replicate sample was composite of leaves from three seedlings. C39, C65, and C75 represent three independent *ShCML44* transgenic tomato lines. WT represents the wild-type AC. Asterisks indicate significant differences between transgenic lines and wild-type. *P < 0.05; **P < 0.01 Dunnett’s multiple comparisons test.

To investigate the role of *ShCML44* in gas exchange attributes under cold stress, physiological analyses including photosynthetic rate (A), transpiration rate (E), intercellular CO_2_ concentration rate (Ci), and stomatal conductance (gs) were determined. Under normal growth conditions, no significant difference was observed in gas exchange attributes for both transgenic lines and wild-type (Fig. 5a–d). Under cold stress (4 °C for 3 d) treatment, all the gas exchange attributes were decreased both in transgenic lines and wild-type plants except the stomatal conductance (gs) was increased in transgenic lines. The transgenic lines demonstrated the significantly higher rate of photosynthetic rate (86.11%), transpiration rate (126.21%), intercellular CO_2_ concentration rate (22.20%), and stomatal conductance (1165.09%) than wild-type plants under cold stress (Fig. 5a–d).

### Drought tolerance of ***ShCML44***-overexpressing plants

**Figure 6:**
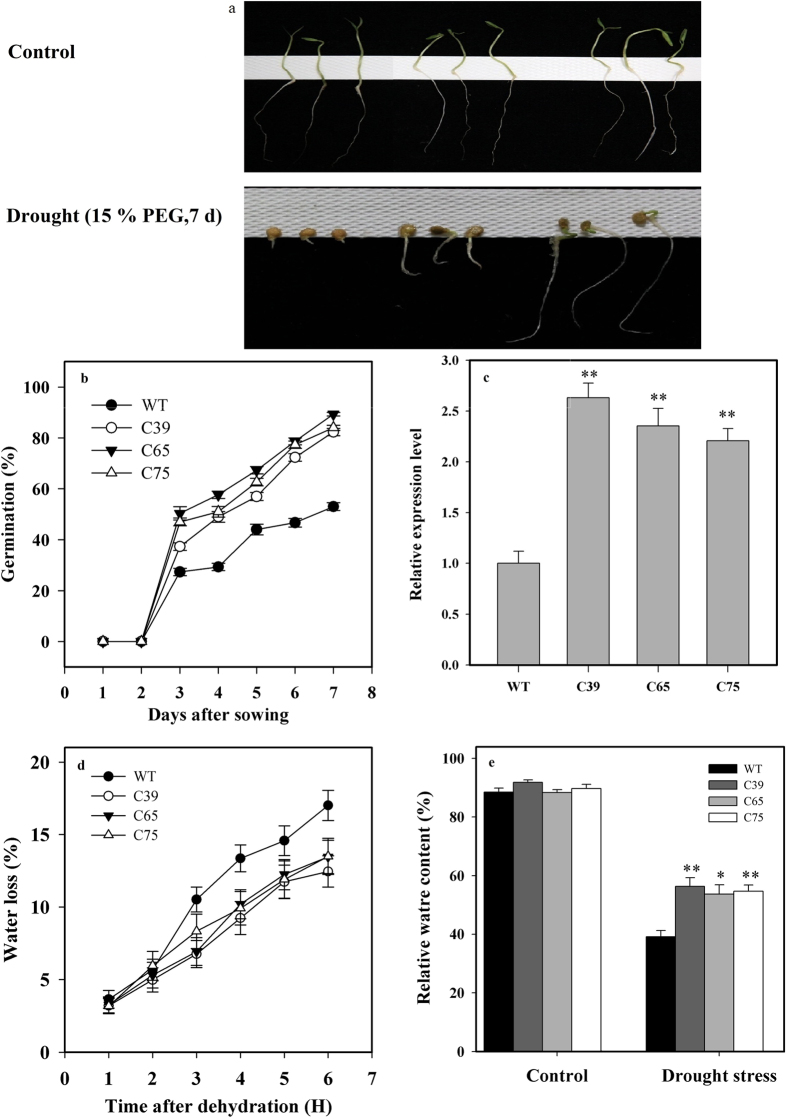
*ShCML44* overexpression plants response towards drought tolerance in tomato. (**a**) Seeding growth of transgenic lines and wild-type plants under control (well-watered) and drought (PEG 15% for 7 days) stress. (**b**) Seed germination of transgenic lines and wild-type plants under drought stress. Seed of transgenic T_2_ lines and wild-type were exposed to 15% PEG- induced drought stress for 7 days. (**c**) *SlCML44* relative expression in wild-type and *SlCML44* and *ShCML44* combined expression in transgenic lines. The relative expression level was normalized to the wild-type expression level, which is considered as 1.0, The EF1α gene was used as an internal control. (**d**) Water loss of the detached leaves from transgenic lines and wild-type plants. Water loss is represented as the percentage of initial fresh weight at each time point. Values are means ± SE of 15 leaves from five plants of each transgenic line or wild-type. Asterisks indicate significant differences between transgenic lines and wild-type. *P < 0.05; **P < 0.01, Dunnett’s multiple comparisons test. Comparison of relative water content (**e**) in leaves of transgenic lines and wild-type plants under normal and drought stress conditions. Data represent means ± SE of three biological replicate samples. Each replicate sample was composite of leaves from nine seedlings. WT represents the wild-type AC. C39, C65, and C75 represent three independent *ShCML44* transgenic tomato lines. Asterisks indicate significant differences between transgenic lines and wild-type. *P < 0.05; **P < 0.01, Dunnett’s multiple comparisons test.

To evaluate the drought tolerance capability of *ShCML44* gene, seedlings of transgenic plants and wild-type were exposed to drought stress (15% PEG for 7 d). The phenotypic responses were observed in terms of germination rate. The transgenic lines exhibited more tolerance than that of wild-type plants under drought stress conditions (Fig. 6a). Germination percentage of transgenic lines was significantly higher (60.80%) than that of wild-type plants (Fig. 6b). The *ShCML44* gene expression was positively associated with increased drought tolerance (Fig. 6c).

No significant difference in leaf RWC was observed in transgenic and wild-type plants under control (well-watered) condition (Fig. 6e). Seedlings of transgenic lines and the wild-type were exposed to 15 d of drought stress treatment. Transgenic lines exhibited significantly higher (40.30%) RWC than that of the wild-type plants (Fig. 6e), indicating the positive role of *ShCML44* in increasing the water retention ability. Water loss assay was performed to further understand the drought tolerance of *ShCML44* overexpressing plants. After dehydration stress, water loss in wild-type plants was increased by 22.73% compared with transgenic lines (Fig. 6d), which confirmed the results of RWC-assay (Fig. 6e).

Exposure of drought stress remarkably effect the morphological growth attributes of tomato seedlings including plant height, number of leaves per plant, leaf length, and leaf width (Fig. S3a–d). Under drought stress, number of leaves per plant, leaf length, and leaf width in transgenic lines were higher than wild-type (Fig. S3a–d).

**Figure 7:**
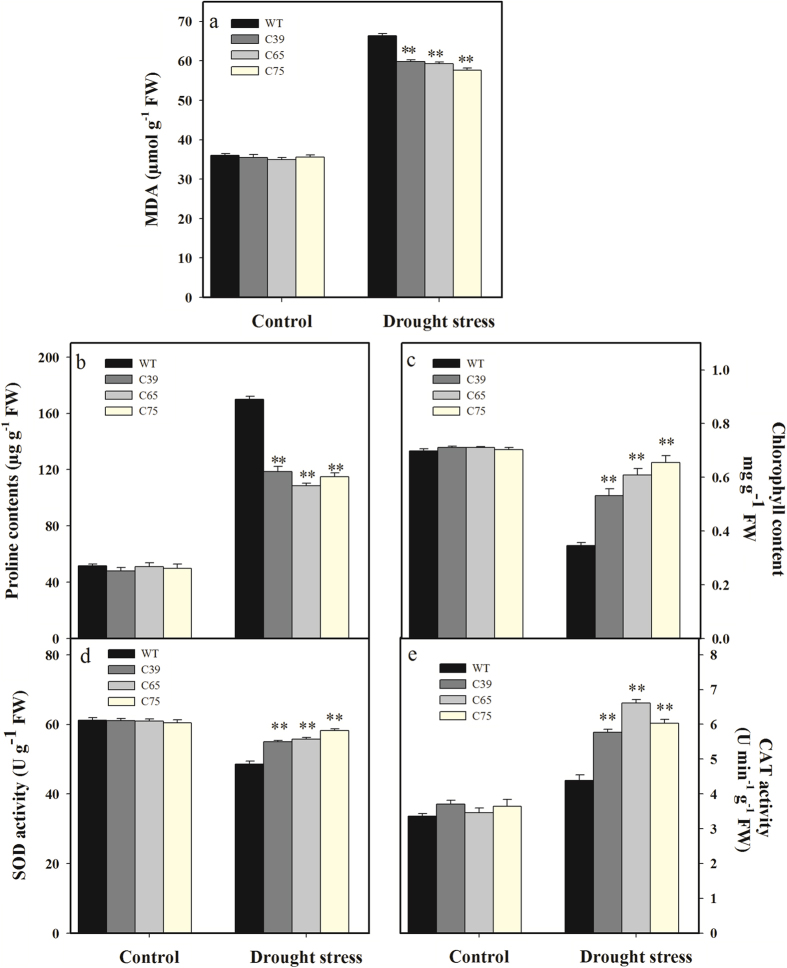
Comparison of enzymatic activity in transgenic and wild-type plants under normal and drought stress conditions. Comparison of MDA content (**a**), proline content (**b**), chlorophyll content (**c**), SOD activity (**d**) and CAT activity (**e**) in the leaves of transgenic lines and wild-type plants under normal and drought stress conditions. Six-week-old seedlings of transgenic T_2_ lines and wild-type were withheld water for 10 successive days (drought stress) or watered every two days (control). The samples shown are representative of six replicates. Values in (**a–e**) represent the means ± SE of three biological replicate samples. Each replicate sample was composite of leaves from twelve seedlings. C39, C65, and C75 represent three independent *ShCML44* transgenic tomato lines. WT represents the wild-type AC. Asterisks indicate significant differences between transgenic lines and wild-type. *P < 0.05; **P < 0.01, Dunnett’s multiple comparisons test.

The MDA, proline and chlorophyll contents were statistically similar in transgenic and wild-type plants under control conditions (Fig. 7a–e). Drought stress was found to enhance the MDA accumulation in tomato seedlings; the transgenic lines showed significantly lower (11%) levels of MDA than wild-type consistent with gene expression level (Fig. 7a). Likewise, exposure of drought stress for 10 d triggered the accumulation of proline content, while, transgenic lines were found to accumulate significantly lower (32.88%) proline content compared with wild-type plants (Fig. 7b). The chlorophyll contents were considerably decreased under the influence of drought stress, however, such a reduction was more for wild type plants. Under drought stress, all the transgenic lines recorded significantly higher (71.07%) chlorophyll contents with respect to wild-type plants (Fig. 7c).

In addition, the antioxidant enzymes (SOD and CAT) activity was determined under drought stress. There were no significant differences in enzymes activity under normal conditions for both transgenic lines and wild-type (Fig. 7d,e). Under drought stress, all the transgenic lines demonstrated significantly higher activities of SOD (15.83%) and CAT (40.09%) than wild-type (Fig. 7d,e), representing higher antioxidant enzymes activity of transgenic lines after drought stress.

**Figure 8:**
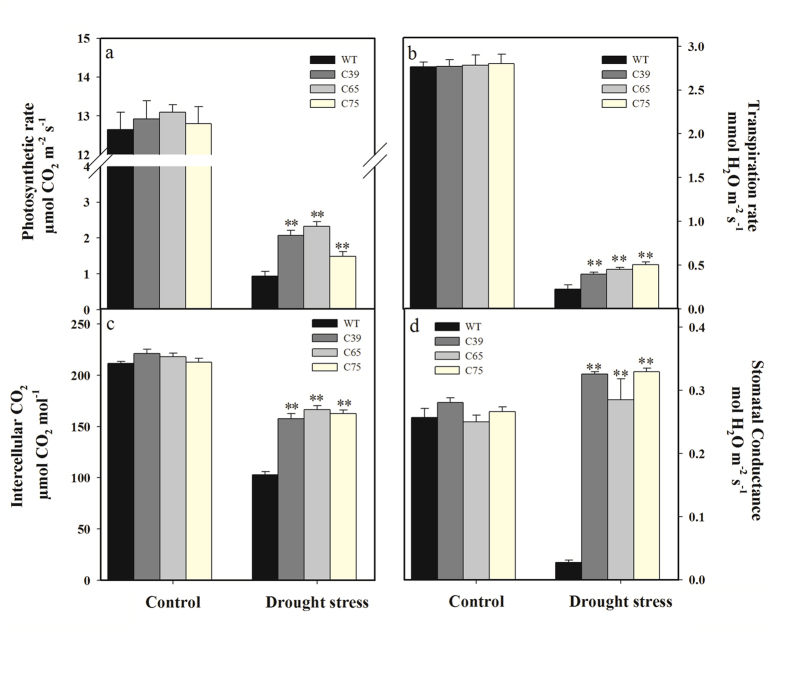
Physiological analysis of transgenic and wild-type plants under normal and drought stress conditions. Comparison of photosynthesis (**a**), transpiration (**b**), intercellular CO_2_ rate (**c**) and stomatal conductance (**d**) in the leaves of transgenic and wild-type plants under normal and drought stress conditions. Six-week old seedlings of transgenic lines (T_2_ generation) and wild-type were treated under cold 4 or 25 °C (control) for 3 d. Values in (**a–d**) represent the means ± SE of three biological replicate samples. Each replicate sample was composite of leaves from three seedlings. C39, C65, and C75 represent three independent *ShCML44* transgenic tomato lines. WT represents the wild-type AC. Asterisks indicate significant differences between transgenic lines and wild-type. *P < 0.05; **P < 0.01 Dunnett’s multiple comparisons test.

The gas exchange attributes including photosynthesis (A), transpiration (E) and intercellular CO_2_ concentration (Ci) were decreased by drought stress, except the stomatal conductance (gs) in transgenic lines. The transgenic lines demonstrated significantly higher photosynthesis (110.06%), transpiration (96.06%), intercellular CO_2_ concentration (57.82%), and stomatal conductance (1060.69%) than wild-type plants (Fig. 8a–d), suggesting the higher gas exchange activity of *ShCML44* transgenic lines than wild-type under drought stress.

### Salinity tolerance of*ShCML44*-overexpressing plants

**Figure 9:**
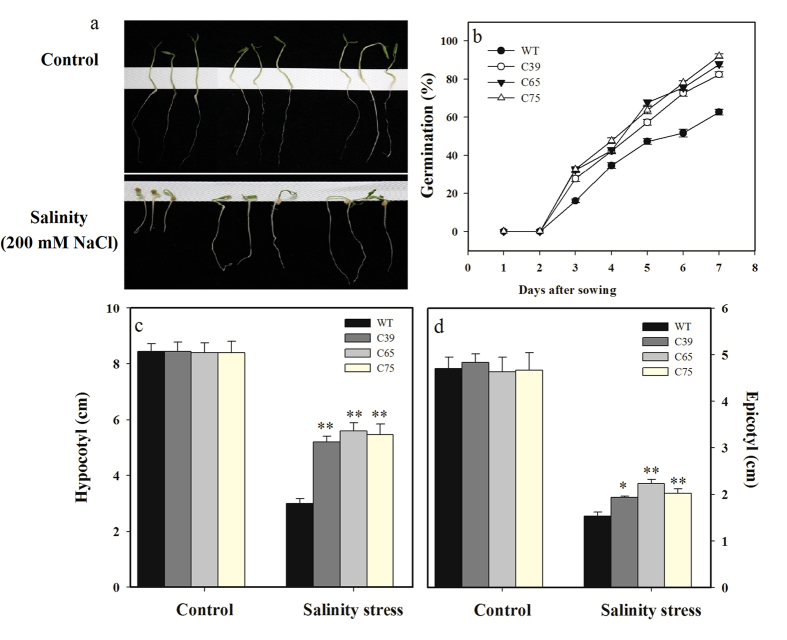
*ShCML44* overexpression plants response towards salinity tolerance in tomato. (**a**) Seeding growth of transgenic lines and wild-type plants under control (0 mM NaCl) and salinity (200 mM NaCl for 7 days) stress. (**b**) Seed germination of transgenic lines and wild-type plants under salinity stress. Seed of transgenic T_2_ lines and wild-type were grown under salinity (200 mM NaCl for 7 days) stress. Comparison of hypocotyl (**c**) and epicotyl (**d**) of transgenic and wild-type plants under normal and salinity stress conditions. Data are means ± SE obtained from 20 seedlings. Asterisks indicate significant differences between transgenic lines and wild-type. *P < 0.05; **P < 0.01, Dunnett’s multiple comparisons test.

To determine the possible role of *ShCML44* gene in salinity tolerance, transgenic lines were evaluated at different growth and developmental stages. During the germination, uniform germinated seeds of transgenic lines, and wild-type was shifted to MS medium plates containing 0 and 200 mM NaCl. After 7 days of salinity stress, transgenic lines showed significantly higher germination (39.35%), hypocotyl length (80.74%), and epicotyl length (34.79%) than wild-type (Fig. 9a–d).

**Figure 10:**
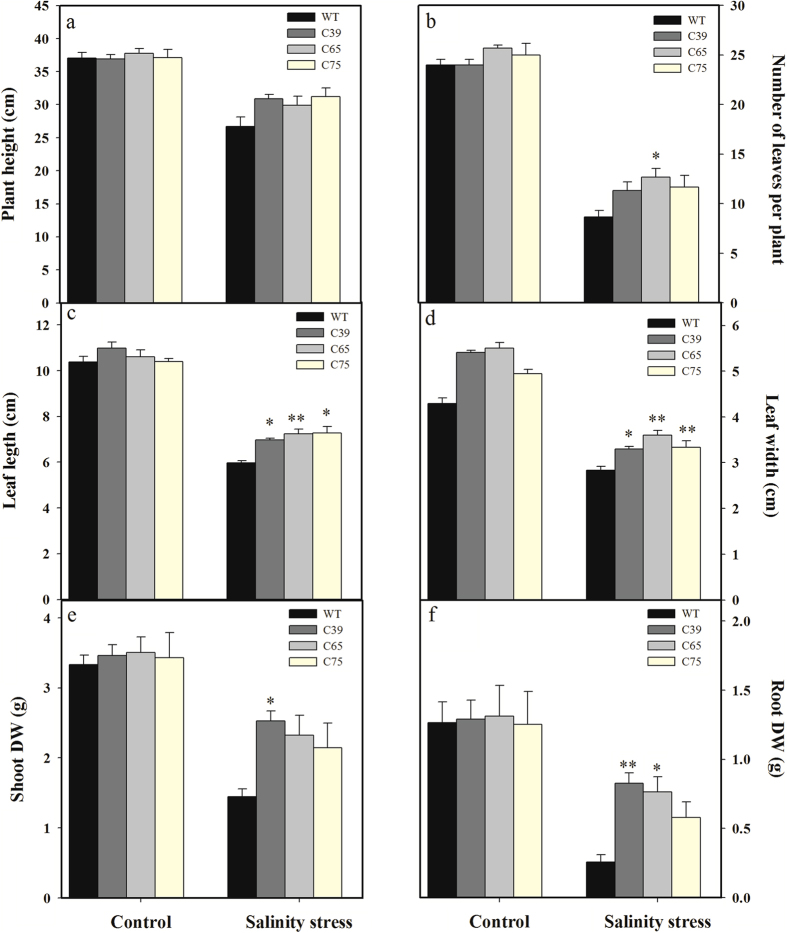
*ShCML44* overexpression plants response towards salt tolerance in tomato. Comparison of plant height (**a**), number of leaves per plant (**b**), leaf length (**c**) leaf width (**d**), shoot dry weight (**e**), and root dry weight (**f**) of transgenic and wild-type plants under normal and salinity stress conditions. Six-week old seedlings of transgenic lines (T_2_ generation) and wild-type were grown with 300 mM NaCl or 0 (control) for 2 weeks. Data are means ± SE obtained from 20 seedlings. WT represents the wild-type AC. Asterisks indicate significant differences between transgenic lines and wild-type. *P < 0.05; **P < 0.01 Dunnett’s multiple comparisons test.

Exposure of salinity stress (300 mM biweekly) hampered the morphological growth attributes of tomato seedlings including plant height, number of leaves per plant, leaf length, leaf width, and biomass accumulation (Fig. 10a–h). Under salinity stress, number of leaves per plant, leaf length, leaf width, and dry biomass accumulation in transgenic lines were increased by 15.03, 37.13, 19.86, 20.53, 60.77, and 189.33%, respectively compared with wild-type (Fig. 10b–h).

**Figure 11:**
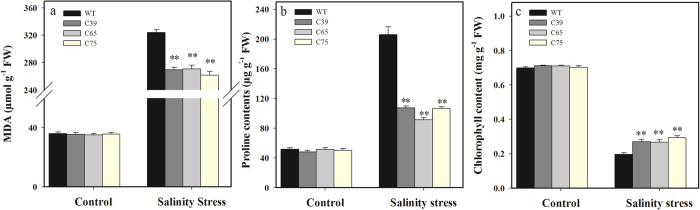
Comparison of MDA (**a**), proline (**b**) and chlorophyll (**c**) contents in transgenic and wild-type plants under normal and salinity stress conditions. Four-week-old seedlings of transgenic T_2_ lines and wild-type were irrigated with NaCl (200 mM) or tap watered (control), and samples were collected after three days. The samples shown are representative of six replicates. Values in (**a–c**) represent the means ± SE of three biological replicate samples. Each replicate sample was composite of leaves from twelve seedlings. C39, C65, and C75 represent three independent *ShCML44* transgenic tomato lines. WT represents the wild-type AC. Asterisks indicate significant differences between transgenic lines and wild-type. *P < 0.05; **P < 0.01, Dunnett’s multiple comparisons test.

No significant difference in MDA, proline, and chlorophyll contents was observed between transgenic and wild-type plants under control (without NaCl) conditions (Fig. 11a–c). Salinity stress (200 mM) was found to enhance the MDA and proline content in tomato seedlings compared with control; the transgenic lines showed significantly lower MDA (17.53%) as well as proline (50.65%) contents than wild-type plants (Fig. 11a,b). Chlorophyll contents were decreased after salinity stress in the transgenic lines and wild-type plants, nevertheless, transgenic lines showed significantly higher (40.57%) chlorophyll content than wild-type plants (Fig. 11c). These findings elucidate the role of *ShCML44* in enhancing the growth and development of transgenic lines under salinity stress.

### Gene expression analysis in ***ShCML44*** overexpressing plants

To get a better understanding regarding the role of *ShCML44* towards stress tolerance, expression analysis of stress-related genes were carried out in transgenic lines, and wild-type plants under control (25 °C) condition by qRT-PCR. Different mechanisms related genes including proline and JA biosynthesis pathways, sugar metabolism, ROS generation and scavenging and abiotic stress related genes were investigated.

**Figure 12:**
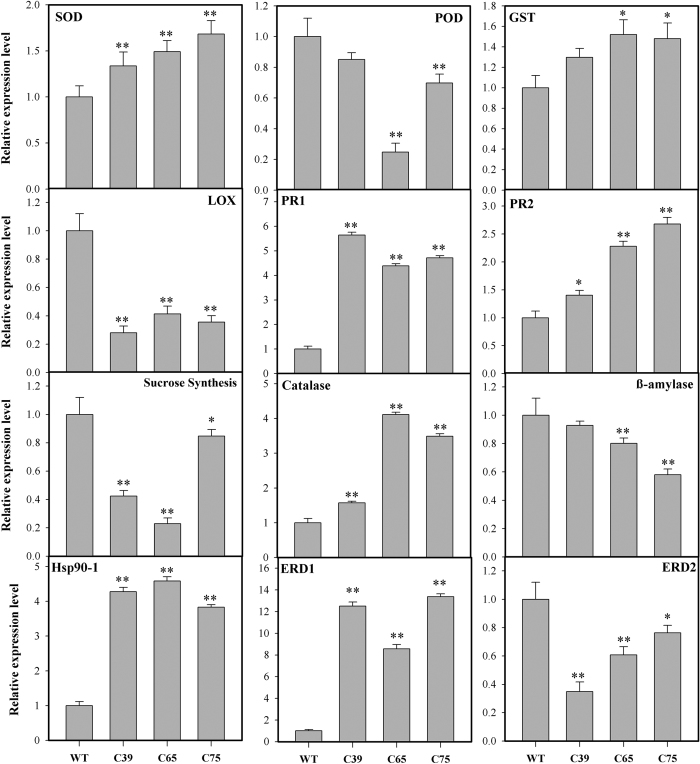
Transcript level of reference genes in *ShCML44* transgenic lines. *ShCML44* transgenic lines showed diverse transcript levels of SOD, GST, Peroxidase, *LOX*, *PR1*, *PR2*, sucrose synthase, Catalase, β-amylase, *Hsp90-1*, *ERD1*, *ERD2* genes. Six-week-old seedlings of wild-type were used to collect samples for RNA extraction. For internal control, EF1α gene was used to normalize expression. Data shown are means ± SE of three biological replicate samples. Each replicate sample was composite of leaves from twelve seedlings. C39, C65, and C75 represent three independent *ShCML44* transgenic tomato lines. WT represents the wild-type AC. Asterisks indicate significant differences between transgenic lines and wild-type. *P < 0.05; **P < 0.01, Dunnett’s multiple comparisons test. SOD: SGN-U213940; GST: SGN-U212747; POD: SGN-U215231; LOX: NM 001247330; PR1: SGN-U215661; PR2: SGN-U215659; Sucrose synthase: SGN-U213118; Catalase: M93719; Beta-amylase: SGN-U213712; Hsp90-1: SGN-U212639; ERD1: SGN-U216637; ERD2: SGN-U228034.

The expression levels of SOD (SGN-U213940), Pathogenesis-related (PR) protein genes (SGN-U215659 and SGN-U215661), Catalase (M93719), molecular chaperone Hsp90-1 (SGN-U212639), and early responsive gene to dehydration ERD1 (SGN-U216637) were significantly up-regulated in all the transgenic lines compared with the wild-type. The glutathione S-transferase GST (SGN-U212747) coding gene was significantly up-regulated in C65 and C75, while remained unchanged in C39 transgenic line, compared with the wild-type. The lipoxygenase gene (*LOX*, NM_001247330), sucrose synthase (SGN-U213118), and ERD2 (SGN-U228034) were significantly down-regulated in all the transgenic lines compared with the wild-type. The POD (SGN-U215231) and β-amylase (SGN-U213712) were only down-regulated in C65 and C75 transgenic lines to a significant level (Fig. 12). These findings suggest the possible involvement of *ShCML44* in transcriptional regulation and/or interaction with other genes to modify transcription factors expression by stress treatments.

## Discussion

In nature, abiotic stress adversely affects the growth and development of tomato regarding germination, vegetative growth, flowering, fruit development and ripening^34^. Previously, traditional molecular breeding techniques have been implemented to overcome the stress losses but minimal progress has been achieved due to the complex mechanism of stress tolerance. Recently, genetic engineering is being considered as an alternative approach to improve the stress tolerance in tomato^6^,^35^. In the past, several stress responsive physiological and biochemical pathways have been documented^4^. For instance, abiotic stress increases the intracellular Ca^2+^ levels to transfer information and stimulate adaptive responses^3^.

Tomato possesses a repertoire of known distinctive proteins including CML (a Ca^2+^ sensor)^14^. Till date, the physiological and molecular functions of CMLs and their involvement in stress response mechanisms is largely unknown^36^. In this study, CML gene family member (*ShCML44*) was isolated from cold-tolerant genotypes (*S. habrochaites*). In our recent investigation^14^, we demonstrated that *SlCML44* is located on chromosome 4, contains 3 EF-hand motifs, 471 bp nucleotide sequence, 156 aa sequence, 4.49 Pls theoretical isoelectric point and 18.01 kDa molecular weight with 30% similarity with *SlCaM* proteins. The EF-hand motif is the most prevalent structure motif identified in Ca^2+^ binding proteins; it expedites the Ca^2+^ sensors to bind Ca^2+^ ions^13^. The presence of three EF-hand motifs and higher identity with *SlCaM44* proteins indicates that ShCML44 might act as Ca^2+^ sensor and function as a transducer in Ca^2+^-mediated signaling pathway in tomato.

In a microarray study, the greater expression of unigene SGN-U227216 encoding *SlCaM44* has been reported in *S. habrochaites* (cold-tolerant) than *S. lycopersicum* (cold-sensitive) after 3 d of cold treatment^31^. In the present study, the qRT-PCR analysis showed significant induction of *ShCML44* under cold (Fig. 2b), drought (Fig. 2c) and salinity (Fig. 2c) stress treatment, which justify the possible involvement of this gene in tomato stress tolerance mechanism. Meanwhile, *ShCML44* tissues specific expression profiling showed diverse accumulation in plant parts (Fig. 2a) which in turn indicates its role in plant growth and development.

CaM and CMLs genes from *S. tuberosum, Nicotiana benthamiana* and *Arabidopsis thaliana* shared identical sequence with *ShCML44* and are responsive to different abiotic stresses including cold, drought and salinity^36^,^37^,^38^. The *ShCML44* functional investigation will provide the further understating to improve homologs crops stress-tolerance. To elucidate the role of *ShCML44*, transgenic tomato lines with *ShCML44* overexpression were generated to analyze the functional performance towards abiotic stress tolerance. The *ShCML44* transgenic lines exhibited cold tolerance with a significant correlation of CML gene expression level (Fig. 3b). These findings are in line with preceding reports^3^,^38^, indicating that overexpression of CML genes correlates with increased ABA sensitivity and stress tolerance. In the present study, the morphological and physiological responses of both wild-type and transgenic lines grown under normal (unstressed) conditions were statistically similar. Contrarily, under stress conditions, the transgenic plants showed higher accumulations of compatible solutes, improved antioxidant enzymes activity, more gas exchange rate, and reduced ROS accumulation, which consequently led to the improved tolerance of transgenic plants to cold, drought, salinity stresses. In plants, abiotic stresses induce the stress-responsive genes expression^3^, coordinately, in the present study, *ShCML44* transgenic plants revealed significant induction of a number of stress-responsive genes (Fig. 12). Taken together, these observations demonstrated that *ShCML44* may be a novel tomato calmodulin-like gene with pleiotropic effects in response to abiotic stress.

Cold stress frequently damage the cell membrane in plants^2^, which can be determined by relative electrolyte leakage and MDA accumulation^39^. In the present study, the cold stress-evoked increments in relative electrolyte leakage and MDA contents were significantly lower in transgenic plants than wild-type (Fig. 3e,f), which indicated that the *ShCML44* transgenic lines negated the cold stress-induced membrane damage and lipid peroxidation. CaM and CMLs have been reported to improve the membrane damage under cold stress treatment. For instance, Yang *et al.* investigated that fish calmodulin was positively regulated in tobacco to inhibit the lipid peroxidation, which results in reduced electrolyte leakage and MDA levels in the transgenic lines than wild-type plants under cold stress^40^. Moreover, it has also been reported that rice calmodulin-like protein *OsMSR2* may improve the stress tolerance and increase the ABA sensitivity of *Arabidopsis* plants^3^.

Reactive oxygen species (ROS) including H_2_O_2_ and superoxide anion (O_2_^−^) induce the membrane lipid peroxidation and function as secondary messengers^41^. Plants contain efficient ROS scavenging defense mechanisms of enzymatic antioxidant including SOD, CAT, POD and APX to protect the oxidative stress-induced cell damage^42^. In this study, *ShCML44* transgenic lines showed higher SOD and CAT activities than wild-type plants under cold and drought stress conditions (Figs 4b,c and 7d,e). The increased expression level of *SOD*, *CAT,* and *GST* genes and decreased expression level of cell wall *POD* gene were observed in transgenic lines (Fig. 12). SOD act as ROS scavenging by dismutating superoxide to H_2_O_2,_ while GST, APX and CAT consequently perform detoxification of H_2_O_2_^41^ and *PODs* contribute in the ROS release or consumption^42^. The SOD has been reported to protect the oxidative stress related membrane damage in tobacco^43^. CAT plays a crucial role in oxidative stress tolerance, such as transgenic tobacco plants with reduced CAT levels exhibited more accumulation of ROS under both abiotic and biotic stresses^44^. The pepper *POD* gene has been revealed to be involved in ROS generation^45^. The ROS scavenging mechanisms decreased the accumulation of ROS in *ShCML44* transgenic lines than the wild-type under cold stress (Fig. 4a). However, higher ROS accumulation in wild-type plants indicates the excessive oxidative stress-induced cell damage. In transgenic lines, the lower oxidative damage was also concomitant with higher chlorophyll contents under stress conditions (Figs 7c and 11c). Indeed, the ShCML44 transgenic lines showed higher abiotic stress tolerance compared to wild-type plants, probably due to preventing photo-oxidation of chlorophyll and acting as an ROS-scavenging protein to limit the lipid peroxidative cell membranes damage under cold, drought and salinity stress.

Ca^2+^ is a critical second messenger and calmodulin-binding proteins including CaM and CMLs are broadly studied Ca^2+^ sensors mediating plant response to abiotic stresses through protecting enzymes and interacting with other downstream target proteins^3^,^24^,^26^,^38^,^46^. For example, *ERD10* and *ERD14* are well-known marker genes sustain normal functions under abiotic stress aggregation to inactivate citrate synthase, alcohol dehydrogenase, lysozyme and firefly luciferase substrates^47^. Liu *et al.* reported that CaM can up-regulate the *hsp26* and *hsp70* marker genes expression in heat-stress, indicating the involvement of CaM in the wheat HS signal transduction pathway^21^. Similarly, CuCOR19, a natural scavenging protein demonstrated protection on oxidative and freezing stress by inactivation of lactate dehydrogenase and catalase in plants^48^. Our findings showed that *ShCML44* overexpressing lines recorded significantly lower proline contents under stress treatment than that of wild-type plants, but no difference was observed in control group (Figs 3h and 7b). Expression analysis also indicated the reduced levels of marker genes related to proline and metabolism (including sucrose synthase and beta-amylase) in transgenic lines than that of wild-type under normal growth conditions (Fig. 12). Rice calmodulin-like (*OsMSR2*) gene improved proline accumulation under stress conditions but no expression alteration was observed in proline biosynthesis genes under normal growth conditions. Probably, *OsMSR2* act as chaperone to improve the proline biosynthetic enzymes stability and stimulates further proline accumulation^3^. In the same way, *ShCML44* may act as chaperone-like protein to strengthen the structure and protect the enzyme’s functions under stress conditions.

Leaf water-retention capacity is strongly related to the stomatal conductance and transpiration rate in plants^49^,^50^. Under stress conditions, our results indicated significantly higher stomatal conductance and transpiration rate in *ShCML44* overexpressing lines than that of wild-type plants (Figs 5b,d and 8b,d). Stomatal conductance and transpiration rate contribute to improving photosynthesis and intercellular CO_2_ concentration rate^51^, our findings demonstrated significantly higher activity of photosynthesis and intercellular CO_2_ rate in transgenic plants compared to wild-type in stress conditions (Figs 5a,c and 8a,c). Furthermore, higher gas exchange attributes play a critical role to improve relative water content by higher root water uptake^50^. Under drought conditions, our findings showed lower leaf water loss rate and improved RWC in transgenic plants than the wild-type (Fig. 6d,e). Previously, in agreement with our results, calcium-sensing receptor (CAS) regulates transpiration, photosynthesis, and stomatal movement by regulating WUE under drought stress in *Arabidopsis*^52^. Similarly, *CML24* (a potential Ca^2+^ sensor gene) was highly induced under drought and salt stress conditions by regulating gas exchange and stomatal mechanisms in *Arabidopsis*^26^. In addition, it is well established that cytosolic Ca^2+^ and ROS play dynamic roles in mediating stomatal closure mechanism in response to water stress and induce various stress adaptation responses^53^,^54^. Stomatal closure is directly related to gas exchange attributes and water-retention capacity^41^. Therefore, the *ShCML44* overexpressing plants with improved gas exchange rate and water-retention ability suggest the involvement of this gene in plant water relations.

It is well stated that CaM and CMLs are most widely studied Ca^2+^ sensors to do hydrophobic and electrostatic interactions with downstream targets proteins. The previous investigations have established that CaM and CMLs act as multifunctional regulatory proteins and play crucial roles in regulating signal transduction or gene expression in plants^38^,^55^. Transcriptome profiling of the potato (*Solanum tuberosum* L.) showed that 19 Calmodulin-like protein were significantly affected by drought stress, which were involved in regulating endogenous hormone biosynthesis and signal transduction pathways^56^. Our data exhibited that *ShCML44* transgenic lines altered the marker genes expression, modulated the antioxidant enzymes, reduced the accumulation of ROS, and improved the gas exchange and water relation attributes under stress conditions. ROS accumulation is crucial for plant complex signaling networks, and excessive accumulation alters the expression of ROS-responsive genes. In this study, JA-responsive gene *LOX* was down-regulated, and two *PRs* were up-regulated in transgenic lines (Fig. 12). Previously, it has been documented that Cam (*SlCaM2*) and CML (*CML37*, *CML38*, and *CML39*) proteins in tomato and *Arabidopsis* were the positive regulator of MeJA and JA^28^,^57^. As expected, our data also showed the rapid and strong induction of *ShCML44* expression after MeJA treatment (Fig. 2c).

Taken together, our results demonstrated that *ShCML44* overexpression plants enhanced the abiotic stress tolerance of tomato plants, possibly due to improved cell membrane integrity, reduced chlorophyll photo-oxidation and accumulation of ROS, lower membrane damage, increased activities of antioxidant enzymes, improved gas exchange and maintenance of plant water relations. All these studied variables were concomitant with higher expression of stress-related genes (Fig. 12). Growing evidence showed that CaMs and CMLs bind to a variety of CaM-binding proteins (CBPs) in plants including kinases, phosphatases, receptors, metabolic enzymes, transcription factors, ion channels and pumps, and cytoskeletal proteins^11^,^19^,^55^,^58^,^59^. Therefore, it is reasonable to conclude that *ShCML44* act as a multifunctional regulatory protein and its functional significance is materialized through the actions of its downstream target proteins in plant responses to abiotic stresses. Further investigations are still required to understand the *ShCML44* functions through binding to downstream target proteins.

## Methods

### Plant material and growth conditions

In the present study, *S. lycopersicum* (cv. Ailsa Craig, AC) and cold-tolerant *S. habrochaites* LA1777 were used. Seeds were sown in plastic pots (10 cm diameter) containing soil, vermiculite, and peat (v/v/v = 1:1:1). The plants were grown at 24–28 °C/20–25 °C (day/night) temperature regime under natural light and 70–80% relative humidity in a greenhouse for 5 weeks. Plant roots, stems, leaves, flowers, and fruits were collected. All the samples were quickly frozen in liquid nitrogen to store at −80 °C until further analysis. For cold and drought stress assay, T_2_ generation homozygous C39, C65, and C75 lines along with wild type (WT) AC were used. For cold and drought treatment, seeds were sown in plastic pots and seedlings were grown under 14-h light/10-h dark photoperiod at 25 ± 2 °C temperature and 60–70% relative humidity in a controlled greenhouse. At the stage of seedlings with six fully expanded leaves, functional leaves from the same position were used in stress tolerance assay.

### Abiotic stress and hormonal treatment

Six-week-old seedlings were shifted to growth chamber under 14-h light/ 10-h dark photoperiod with 25 ± 2 °C temperature with 200 μmol m^−2^s^−1^ light intensity. For stress and hormonal treatment, seedlings with six fully expanded leaves were used after five days of adaptation period. Various treatments including drought, salt, abscisic acid (ABA), methyl jasmonate (MeJA) and ethephon (Eth) were carried out under the same conditions as above. For cold stress, seedlings of LA1777 and AC were transferred to a growth chamber at 4 °C. For drought stress, seedlings of LA1777 were uprooted, thoroughly washed and dehydrated on filter papers. For salt stress, seedlings of LA1777 were irrigated with 200 mM NaCl. For hormonal treatment, seedlings were sprayed with 100 μM ABA, 100 μM MeJA or 1% (v/v) Eth. Seedlings mock-treated with water were used as the control. Functional leaves from the same position were collected at indicated (0, 1, 3, 6, 9, and 12 h) time points after the treatment. For all the treatments, three biological samples were collected and frozen immediately in liquid nitrogen to store at −80 °C for RNA isolation.

### Isolation and sequence analysis of ***ShCML44***

TriZol reagent (Invitrogen, USA) was used to isolate total RNA. To synthesize the first-strand cDNA, MMLV reverse transcriptase (Toyobo, Japan) was used as per manufacturer’s instructions. By using the leaf cDNA of LA1777, the full-length open reading frame (ORF) of *ShCML44* was amplified. The sequence specific primer (*ShCML44*, Table S1) were designed according to *S. lycopersicum* full-length cDNA LOC101245247 (designated as *SlCML44*, GenBank accession no. XM_004237307.2). To clone the amplified PCR product, pEASY-blunt cloning vector (TransGen, China) was used and consequently the recombinant clones were sequenced to identify the correct sequence. For gene prediction online SoftBerry tool (http://linux1.softberry.com/berry.phtml) and for sequence alignment ClustalW2 (http://www.ebi.ac.uk/Tools/msa/clustalw2/) were used.

### Plant transformation and molecular characterization of transgenic tomato

The pEASY-blunt cloning vector containing the correct and complete ORF was double digested using *Kpn*I and *Xho*I. The recovered fragment was ligated into the plant binary vector pMV digested/cleaved with *Kpn*I and XhoI. The resulting construct allowing the gene to be driven by the cauliflower mosaic virus (CaMV) 35S promoter. The construct was transformed into *Agrobacterium* strain C58 by electroporation and used for transformation on tomato AC *via* the standard leaf disk protocol^60^. Primary tomato plants (T_0_) were selfed, and T_1_ seeds were collected. These transformed plants were confirmed by PCR using CaMV 35S promoter specific forward and *ShCML44* reverse primer (Table S1). Segregation analysis was performed to select homozygous lines as described by Pino *et al.*^61^. For expression analysis of CML transgenic lines, quantitative real-time PCR (qRT-PCR) was performed. For further study, three independent homozygous lines (C39, C65, and C75) of *ShCML44* with higher expression levels were selected.

### Abiotic stress tolerance assays

#### Cold

To determine the cold tolerance at an early stage, pots with the seedlings of WT and transgenic lines (C39, C65, and C75) were moved into a controlled cold chamber at 4 °C. After 7 days of treatment, photographs were taken, survival and germination percentage were determined.

For cold tolerance evaluation after 3 days of treatment, the fully expanded second and third leaves from the top were collected and immediately frozen in liquid nitrogen to store at −80 °C. These samples were further used to determine the gene expression, membrane permeability, MDA, proline and antioxidant enzymes activities. The MDA content, proline content, electrolyte leakage, and the activities of antioxidant enzymes (SOD, POD, CAT, and APX) were determined according to the methods described in Liu *et al.*^6^. This experiment was repeated, and 20 plants of each line were used each time. To determine the gas exchange attributes and measure the photosynthetic system under cold stress, two months old plants were exposed to cold stress for one week and the total chlorophyll, photosynthetic rate (A), transpiration rate (E), intercellular CO_2_ concentration rate and stomatal conductance (gs) were measured. The detailed methodology for the measurements of gas exchange attributes and total chlorophyll content is described in Zhao *et al.*^50^.

#### Drought

To evaluate the drought tolerance at an early stage, seeds of WT and transgenic lines were sown on MS medium plates containing 0 and 15% PEG solution. After 7 days of treatment, seedlings were photographed, and germination percentage was determined.

The drought tolerance experiment, seedlings of WT as well as transgenic lines were divided into two groups: drought treatment (DT) and control. DT group was kept unwatered for 10 days, whereas, control group watered after every alternate day. After 10 days of treatment, fully expanded leaves of the control and stressed plants from the top were collected and ground in liquid nitrogen and stored at −80 °C. These samples were further used to measure the MDA, proline, and antioxidant enzymes activities as well as to determine relative water content (RWC). The RWC was determined according to the method described by Shekhawat *et al.*^62^. For water loss determination, fully expanded leaves from the top of WT and transgenic lines were harvested and placed on filter paper under room temperate and white florescent light. These leaves were weighed periodically at an interval of 30 min up to 6 h. Water loss was expressed as a percentage of the control. Gas exchange attributes and photosynthetic pigments were determined after exposure of two months old plants to drought stress for three weeks.

#### Salinity

To perform the salt assay at germination stage, seeds of WT and transgenic lines, sown on MS medium plates containing 0 and 200 mM NaCl. After seven days of treatment, seedlings were photographed, germination percentage and seedling length was determined. Four weeks old seedlings grown in pots, were irrigated with NaCl (200 mM) and samples were collected after three days of treatment to measure MDA and proline contents. To evaluate the salinity effect on growth, six weeks old plants of WT and transgenic lines were exposed to 300 mM NaCl biweekly. The control plants were supplied with tap water. Morphological attributes including plant height, number of leaves, leaf width, leaf length and plant biomass, were measured. In another detached leaf salt assay, leaf dishes (1 cm diameter) from top fully expanded leaves were floated in 200 mM NaCl concentrations. These samples placed under white light at 25 ± 2 °C. After 72 hours of treatment total chlorophyll content were measured. This assay was repeated twice.

### Expression analysis by qRT-PCR

The qRT-PCR was performed to analyze the expression pattern of *SlCML44* gene in different plant tissues of LA1777. Further, CML (*ShCML44*) gene expression under stress treatments was quantified. Moreover, expression levels of selected novel genes in transgenic lines were also evaluated. LightCycler 480 instrument (Roche Diagnostics, Basel, Switzerland) and SYBR^®^ Premix Ex Taq^TM^ (TaKaRa, China) was used according to the supplier’s instructions to perform real-time PCR (qRT-PCR). The thermo-cycling conditions for real-time PCR were as follows: 95 °C for 30 s; 40 cycles of 95 °C for 5 s, and 60 °C for 25 s. All the experiments were performed in triplicates. The identical qRT-PCR primer pair for *SlCML44* and *ShCML44* was used. Tomato actin gene (GenBank ID: BT012695) was used as an internal control. Real-time PCR data was analyzed using 2^−ΔΔCT^ method^63^. The primers were designed by Primer3 (http://frodo.wi.mit.edu/primer3) and listed in Table S1.

### Statistical analyses

Data was analyzed statistically by using GraphPad Prism 6 and SigmaPlot 12. CML genes relative expression under abiotic stress and phytohormones are the mean ± SE of three biological replicate samples. Composite of leaves from three individual seedlings considered as a replicate sample. Expression level at control time point (0 h) was defined as 1.0 for each treatment. Expression level difference between LA1777 and AC under cold stress, and each treated time point (1, 3, 6, 12, or 24) and control (0 h) under stress and phytohormones were analyzed statistically using the Student’s t-test and Dunnett’s multiple comparisons test, respectively. The data for MDA, total chlorophyll, proline and antioxidant enzymes activities are mean ± SE of three biological replicates. Each replicate sample was a combination of leaves from twelve seedlings. RWC and leaf water loss are means ± SE collected from 12 leaves. The values for fresh weight, primary root length, and lateral root number are means ± SE gained from 15 seedlings. Gas exchange attributes were determined by sampling three leaves of each plant, and 10 plants were sampled from the transgenic lines and the WT plants, respectively. A significant difference between each transgenic line and wild-type was distinguished by Dunnett’s multiple comparisons test. Asterisks * and ** indicate significant differences at P < 0.05 and P < 0.01, respectively.

## Additional Information

**How to cite this article**: Munir, S. *et al.* Overexpression of calmodulin-like (*ShCML44*) stress-responsive gene from *Solanum habrochaites* enhances tolerance to multiple abiotic stresses. *Sci. Rep.* **6**, 31772; doi: 10.1038/srep31772 (2016).

## Supplementary Material

Supplementary Information

## Figures and Tables

**Table 1:** Putative cis-elements identified in *ShCML44* promoter.

Sequence	Function	Element number
ATTAAT	Involved in light responsiveness	3
CGTCA	MeJA-responsiveness	1
ATTTCAAA	Ethylene-responsive element	1
AAAAAATTTC	Heat stress responsiveness	2
TATCCCA	Involved in gibberellin-responsiveness	1
GAGAAGAATA	Involved in salicylic acid responsiveness	1
CGTGG	Auxin responsive element	1
TCCAAGTATA	Response to wounding and tensile stress	1
AAATTTCCT	Wound-responsive element	1
CAANNNNATC	Involved in circadian control	1
ACGTG	Dehydration responsive element	2
MACGYGB	Calcium responsive site	1
ACGT	Responsive to dehydration	6
AAACAAA	Anaerobically induction element	2
TGTCTC	Auxin response factor binding site	1
TGACG	Auxin and salicylic acid responsive site	2
CCAAT	Heat shock responsive	5
TATTAG	Cytokinin responsive	2
GTAC	Copper- and oxygen-responsive	10
AWTTCAAA	Ethylene responsive element	2
TAACAAR	GA-responsive element	1
GAAAAA	Pathogen- and salt-induced	5
CCGAC	Cold-, drought- or ABA-induced	1
WAACCA	Dehydration responsive	1
TAACAAA	GA-regulation element	1
CANNTG	ABA, cold, and dehydration responsive	10
ACTTTA	Auxin responsive element	1
AACGTGT	Response to wounding and tensile stress	1
AACCAA	Phytochrome regulation element	1
CATGCA	ABA-responsive element	1
AACGTG	Responsive to wounding and jasmonate	2
TATCCA	Response to gibberellin, sugar	1
TGACY	Responsive to wounding	6
TGAC	Gibberellin repression, pathogen response	13
