# Supplementary material for: Overexpression of calmodulin-like (ShCML44) stress-responsive gene from Solanum habrochaites enhances tolerance to multiple abiotic stresses
Source: Sci Rep. 2016 Aug 22;6:31772. doi: 10.1038/srep31772 (PMC4992891; doi:10.1038/srep31772)
Supplement: Supplementary Information [file srep31772-s1.pdf]

Supplementary material

Overexpression of calmodulin-like (*ShCML44*) stress-responsive gene from *Solanum habrochaites*, enhances tolerance to multiple abiotic stresses

Shoaib Munir[1], Liu Hui[2], Xing Yali[1], Saddam Hussain[3], Bo Ouyang[1], Yuyang Zhang[1], Hanxia Li[1], Zhibiao Ye[1]*

[1]Key Laboratory of Horticultural Plant Biology (Ministry of Education), College of Horticulture and Forestry Sciences, Huazhong Agricultural University, Wuhan 430070, China

[2]Key Laboratory of Biology and Genetic Resources of Rubber Tree, Ministry of Agriculture, Rubber Research Institute, Chinese Academy of Tropical Agricultural Sciences, Danzhou 571737, China

[3]College of Resources and Environment, Huazhong Agricultural University, Wuhan 430070, China

*Corresponding author: Zhibiao Ye

E-mail: zbye@mail.hzau.edu.cn

Fax: +86-27-87282010

Table S1. List of primer sequences.

Gene	Forward primer	Reverse primer
**For gene amplification and qRT-PCR**		
ShCML	TCATGTCTCCGATCAACTCAAT	TTGGTAGATTGTAAGCGGGATT
ß-actin	ATGGCAGACGGAGAGGATATTCA	AGACGGAGAATGGCATGTGG
EF1a	CGTGGTTATGTTGCCTCAAA	ACAGCAATGTGGGAAGTGTG
**For promoter amplification**		
PCML	TCGGAGGCAAAATTAAGGAA	TGACGCGAAATAATGATGCT
**For transgenic plants identification**		
ShCML	TCATGTCTCCGATCAACTCAAT	TTGGTAGATTGTAAGCGGGATT
CaMV35S	ACGCACAATCCCACTATCCTTC	
**For qRT-PCR**		
CML	ATGTCTCCGATCAACTCAAT	TACCAAGTAGCAACTCTAGC
SOD	TTTCTGGGAAGCAAATCGTC	TTGGGCAATGAAGAAGAAGC
Peroxidase	CTTGCCCTAATGCTCTCACC	GCATCACAACCCTGAACAAA
GST	GCAAGCCCATTTGTGAGTCT	TGCTGACCCCTTATCATCG
LOX	TCATTTTCCCCTGGCAAGTA	TGGTGCATTTGGATCTTCCT
PR1	GGATTGACACCAGCAGAATC	CCATGTAACCCCAATGCCTA
PR2	ATGGGCTGAAGGATCAGTTG	GACACTCTTCTTGAACTTTGTTGG
Catalase	TCCCAGTTAATGCTCCCAAG	CTCAGCAGGACGACAAGGAT
sucrose synthase	GTGGAGACCGAAGAAAGGAA	GGGAAGAAATCCATCGGAAC
β-amylase	GCATTCGTGCCAACTTATCTT	GATTTCCACTCCGTGCTTTC
Hsp90-1	GCACTTCTCTGTTGAAGGTCAG	ATGAACACACGGCGAACATA
ERD1	CAGAACTGGCCTCAAGCAC	AGCATGGCAGATTCAGATCC
ERD2	ATTTTGAGTGGCGAGGGTAA	TTGGGATAGTCGTGTTTCTGG

Supplementary Figures legend

Figure S1. Alignment of *ShCML44* nucleotide sequence with sequences from *S. lycopersicum* (*SlCML44*) and *S. pennellii* (*SpCML44*). Multiple sequence alignments were conducted using ClustalW2.

Figure S2. Phylogenetic analysis of *Solanum habrochaites* (*ShCML44), S. lycopersicum* (*SlCML44*) and *S. pennellii* (*SpCML44*) proteins with other species CML proteins.

Figure S3. *ShCML44* overexpression plants response towards drought tolerance in tomato. Comparison of plant height (a), number of leaves per plant (b), leaf length (c) leaf width (d), of transgenic and wild-type plants under normal and drought stress conditions.

Figure S1

```
SlCML                    ATGTCTCCGATCAACTCAATTAATTTGTCAAAAATCTTCTCAAAGCTTGACAAGAATGGT
SpCML                    ATGTCTCCGATCAACTCAATTAATTTGTCAAAGATCTTCTCAAAGCTTGACAAGAATGGT
ShCML                    ATGTCTCCGATCAACTCAATTAATTTGTCAAAAATCTTCTCAAAGCTTGACAAGAATGGT
                         ********************************.***************************

SlCML                    GATGGCCTTGTGTGTCTTGATGAGTTAAAGGGATTTCTTGATACAATAGGAATTATTGCA
SpCML                    GATGGCCTTGTGTGTCTTGATGAGTTAAAGGGATTTCTTGATACAATAGGAATTATTGCA
ShCML                    GATGGCCTTGTGTGTCTTGATGAGTTAAAGGGATTTCTTGATACAATAGGAATTATTGCA
                         ************************************************************

SlCML                    AGCCAAGAGGAGCTAGAGTTGCTACTTGGTAAAACAAGCCTAGACTCCATTGATTTTTTC
SpCML                    AGCCAAGAGGAGCTAGAGTTGCTACTTGGTAAAACAAGCCTAGACTTCATTGATTTTTTC
ShCML                    AGCCAAGAGGAGCTAGAGTTGCTACTTGGTAAAACAAGCCTAGACTCCATTGATTTTTTC
                         ********************************************** *************

SlCML                    TTCTTCTATGATGCTATCACAAAGGCAAATATTAATAAAGGTAGCAATTATAAGCACGAG
SpCML                    TTCTTCTATGATGCTATCACAAAGGCAAATATTAATAAAGGTAGCAATTATAAGCACGAG
ShCML                    TTCTTCTATGATGCTATCACAAAGGCAAATATTAATAAAGGTAGCAATTATAGACACGAG
                         ****************************************************..******

SlCML                    GATCGAGAAAATGTTTTCTTGGAAAATGACCTACGTAAAGTGTTTAGAGTATTCGATTTA
SpCML                    GATCGAGAAAATGTTTTCTTGGAAAATGACCTACGTAAAGTGTTTAGAGTATTCGATTTA
ShCML                    GATCGAGAAAATGTTTTCTTGGAAAATGACCTACGTAAAGTGTTTAGAGTATTCGATTTA
                         ************************************************************

SlCML                    AACGAGGACGGATTCATATGTTGTGAGGAGCTGCAAAGAGCATTGTCAAGATTAGGATTG
SpCML                    AACGAGGACGGATTCATATGTTGTGAGGAGCTGCAAAGAGCATTGTCAAGATTAGGATTG
ShCML                    AACGAGGACGGATTCATATGTTGTGAGGAGCTGCAAAGAGCATTGTCAAGATTAGGATTG
                         ************************************************************

SlCML                    TGGGATGAACAATGTGGGAAAGATTGTAAGAGTATGATCAATGTTTATGACAAAAATTTA
SpCML                    TGGGATGAACAATGTGGGAAAGATTGTAAGAGTATGATCAATGTTTATGACAAAAATTTA
ShCML                    TGGGATGAACAATGTGGGAAAGATTGTAAGAGTATGATCAATGTTTATGACAAAAATTTA
                         ************************************************************

SlCML                    GATGGAAAACTTGATTATGAGGAGTTTAAAGACATGATGTTTGATAATTAA
SpCML                    GATGGAAAACTTGATTATGAGGAGTTTAAAGACATGATGTTTGATAATTAA
ShCML                    GATGGAAAACTTGATTATGAGGAGTTTAAAGACATGATGTTTGATAATTAA
                         ***************************************************
```

Figure S2

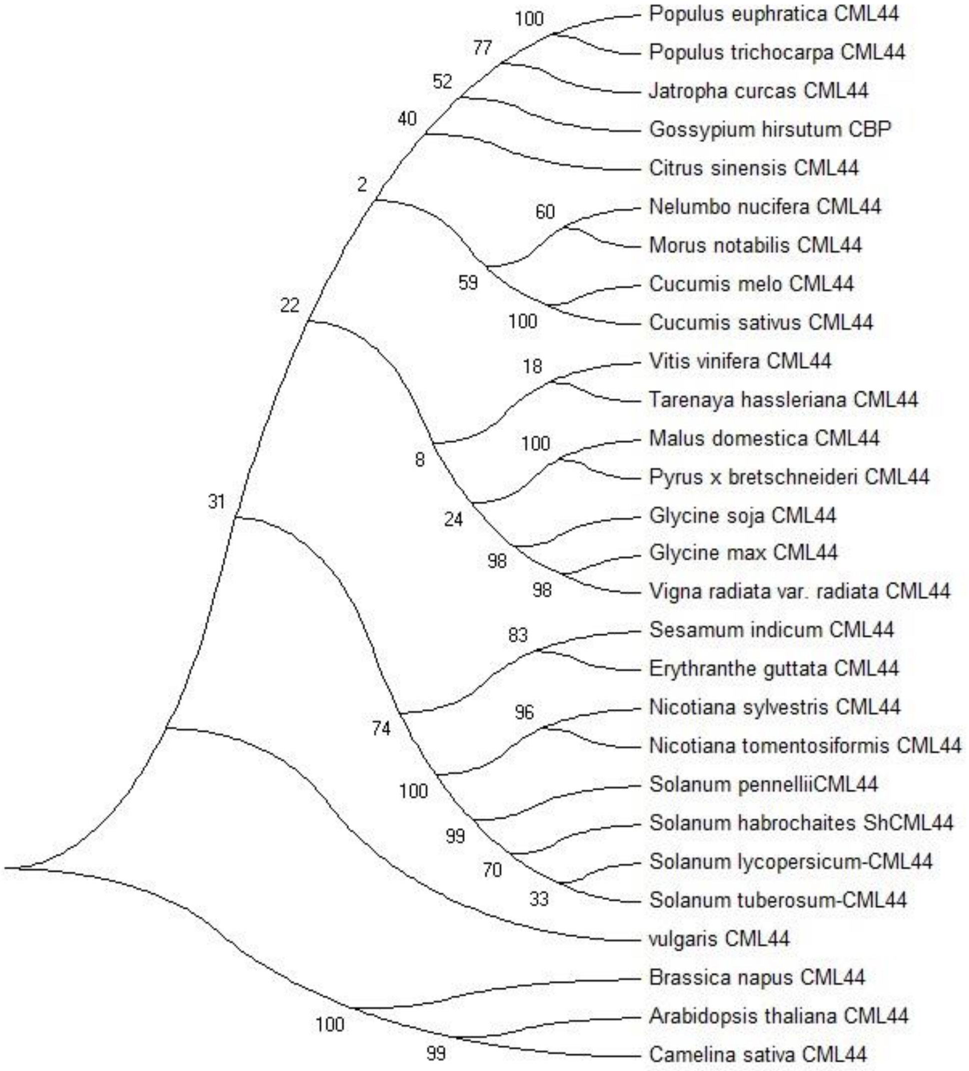
Populus euphratica CML44
Populus trichocarpa CML44
Jatropha curcas CML44
Gossypium hirsutum CBP
Citrus sinensis CML44
Nelumbo nucifera CML44
Morus notabilis CML44
Cucumis melo CML44
Cucumis sativus CML44
Vitis vinifera CML44
Tarenaya hassleriana CML44
Malus domestica CML44
Pyrus x bretschneideri CML44
Glycine soja CML44
Glycine max CML44
Vigna radiata var. radiata CML44
Sesamum indicum CML44
Erythranthe guttata CML44
Nicotiana sylvestris CML44
Nicotiana tomentosiformis CML44
Solanum pennelliiCML44
Solanum habrochaites ShCML44
Solanum lycopersicum-CML44
Solanum tuberosum-CML44
vulgaris CML44
Brassica napus CML44
Arabidopsis thaliana CML44
Camelina sativa CML44
100
77
52
40
2
60
59
100
22
18
100
8
24
98
98
31
83
96
74
100
99
70
33
100
99

Figure S3

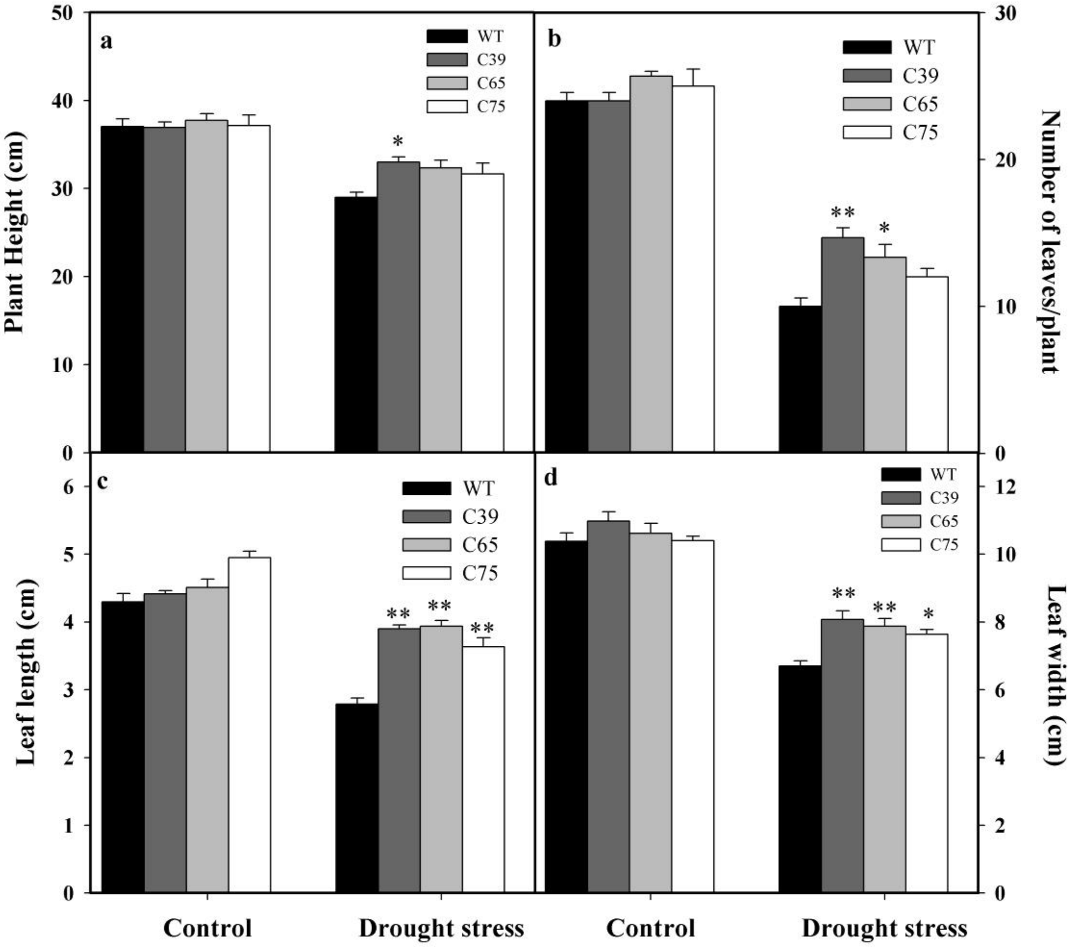

a
b
c
d
WT
C39
C65
C75
Plant Height (cm)
Number of leaves/plant
Leaf length (cm)
Leaf width (cm)
Control
Drought stress
